# Adversity of prolonged extreme cold exposure among adult clients diagnosed with coronary artery diseases: a primer for recommending community health nursing intervention

**DOI:** 10.1002/nop2.111

**Published:** 2017-12-23

**Authors:** Eladio Martin S. Gumabay, Raquel C. Ramirez, Judy Mae M. Dimaya, Mae M. Beltran

**Affiliations:** ^1^ Center for Health Research and Development University of Saint Louis Tuguegarao City Cagayan Valley Philippines; ^2^ University of Saint Louis Tuguegarao City Cagayan Valley Philippines

**Keywords:** climate change, cold adversities, coping, Coronary Artery Disease (CAD), extreme cold

## Abstract

**Aim:**

This research study explored the lived experiences of adults diagnosed with Coronary Artery Disease (CAD) when exposed to a prolonged period of extreme cold.

**Design:**

This research study utilized descriptive qualitative research design.

**Methods:**

Face‐to‐face interview sessions with audio recording were conducted. There were 30 informants who participated in the study. Descriptive phenomenology with Colaizzi's method of data analysis was used.

**Results:**

Results revealed three themes, namely: (i) elucidating cold exposure; (ii) challenges of cold exposure; and (iii) translating adverse exposure to self‐management. The results further revealed the significance of nursing health care especially to health promotion, disease prevention and health restoration especially in community setting.

**Conclusion:**

In conclusion, manifestations of CAD are triggered when exposed to a prolonged period of extremely low environmental temperature.

## 1 INTRODUCTION

Climate change threatens the health status of all individuals through extreme temperatures, both the hot and the cold weather condition (Vardoulakis et al., 2014) presented that prolonged and extreme cold exposure of adults contributes to the development and aggravation of coronary artery diseases (CADs).

Adult individuals diagnosed with CADs and living in the coastal area at the northern part of the Philippines are at high vulnerability to the changes in weather condition, specifically, during prolonged cold season. This insight of the researchers is undergirded by the study of Hajat et al. (2015) and Masato et al. (2015) postulate that a long‐standing problem exists relevant to extreme cold exposure in coastal and rural areas.

The most affected people are elders, young adults and children (Davídkovová, Plavcová, Kyncl, & Kyselý, 2014) but people with existing cardiovascular disease and comorbid illnesses have greater risk from prolonged extreme cold exposure (Lavigne et al., 2014).With the threats currently posed by climate change to the cardiovascular system of adult clients, the researchers explore the lived experiences of adult clients diagnosed with CAD on the adversities of prolonged extreme cold exposure.

## 2 BACKGROUND

Climate change is the variation in weather pattern (high and low temperature in long period of time) in a specific region, relatively used to describe and define the history of human civilization usually featured through social network system and Internet enhanced with the advancement of modern technology which effectively increases the general public awareness (Lineman, Yuno, Ji, & Gea‐Jae, 2015; Samson, Berteaux, McGill, & Humphries, 2011; Vardoulakis et al., 2014).

Nowadays, climate change is a major scientific issue and in the future decades because of its severe adverse effect disturbing the smooth flow of socioeconomic, industrial and agricultural aspect (Byravan & Rajan, 2015; Ryall, 2014; Singh & Purohit, 2014; Wergen, Hense, & Krug, 2014). It increases the global temperature in long‐term trend dramatically threatens the human's well‐being particularly people's livelihood, safety and security, likewise a danger to animals, species and to the health of global ecosystem (Bradley et al., 2012; Lineman et al., 2015; Schuldt & Roh, 2014; Zheng, Ding, Hao, & Ge, 2012). Hamilton, Hartter, Lemcke‐Stampone, Moore, and Safford (2015) mainly expressed that climate change has a great impact to every individual according to age, sex and education making it as a vital global problem to look forward for a critical solution.

Epidemiological studies confirmed that multidimensional catastrophic phenomenon is a growing public health concern due to strong association in rampant morbidity and mortality worldwide chiefly the increase frequency of extreme weather (Lavigne et al., 2014; Schuldt & Roh, 2014; Singh & Purohit, 2014). In Europe and some parts of America, winter season strikes longer compared to hot weather possibly due to seasonal temperature changes leading to outnumbered morbidity and mortality. This is mostly attributed to respiratory diseases due to infections and influenza, diabetes, cerebrovascular diseases and cardiovascular diseases (CVD). Likewise, CAD have the largest death rate during cold season (Hajat et al., 2015; Marti‐Soler et al., 2014; Masato et al., 2015; Wergen et al., 2014; Xie et al., 2013; Zeka, Browne, McAvoy, & Goodman, 2014).

Basically, extreme cold that lasts for many months is remarkably hazardous to the community that can cause harm affecting vulnerable people especially elders, children, women and susceptible patients including those with comorbidities such as cardiovascular diseases, diabetes, respiratory and CAD which has the highest risk among all (Davídkovová et al., 2014; Lavigne et al., 2014; Masato et al., 2015; Ryall, 2014; Xie et al., 2013; Zhaoxing, Shanshan, Jinliang, Jaakkola, & &Yuming, 2012).

Indeed, based on the findings of numerous studies, extreme cold distresses most people living in rural areas, coastal villages and shorelines (Byravan & Rajan, 2015; Masato et al., 2015; Zhou et al., 2014). However, Hamilton et al. (2015) assumed that place‐to‐place variation in daily weather temperatures aggravates the severity of the atmospheric condition and actually associated to the local structure.

On the other hand, there is a complex relationship of people's adaptive ability to climate change or the constant environmental stimulus. For example, extreme low temperature that distracts the individual's well‐being and functioning. This is theoretically supported by the Adaptation Theory of Sister Callista Roy depicting that a person is a biopsychosocial being obliged to adapt environmental stimuli to maintain the physical and physiological integrity of human system (Bowlin, McLeer, & Danielson‐Francois, 2014; Fawcett & Tulman, 1990; Masato et al., 2015; Tansey & Johnson, 2015; Xie et al., 2013). Adaptation theory is the foundation in nursing health care essential in delivering excellent and systematic wellness through indispensable health promotion, disease prevention and health restoration highlighting the valued goals of community health nursing which are physical, group identity, role function and interdependence concept (Bradley et al., 2012; Dixon, 1999; Fawcett & Tulman, 1990).

This research study explored the adversities of prolonged cold exposure of adult clients diagnosed with CAD. This presents interpretations, insights and lived experiences of adult clients suffering from cardiovascular disease specifically when lengthy exposed to intense cold. In addition, various managements will be described and narrated according to individual's unique experiences. Foremost, the research study is of significance to the community in collaboration with healthcare institutions and immersed health professionals to strengthen interventions and to supplement dynamic information pertaining to prolonged extreme cold exposure relative to cardiovascular disease.

Specifically, this research study addressed the following major inquiries:

- What are the perceptions of adult clients diagnosed with CAD relevant to prolonged extreme cold exposure?
- What are the adverse health effects of prolonged extreme cold exposure of adult clients diagnosed with CAD?
- How do adult clients diagnosed with CAD manage the adverse health effects of prolonged extreme cold exposure?

## 3 THE STUDY

### 3.1 Research Design

The researchers used descriptive qualitative phenomenological design to determine and describe the various adversities of prolonged extreme cold exposure among adults diagnosed with CAD. This study explored through collecting and examining the people's comprehensive description, perception and interpretation regarding the nature of lived experiences in a certain phenomenon (Ingham‐Broomfield, 2015; Sampa, 2012). The approach was initially broad focused and process oriented in gathering accurate and rich information (Malagon‐Maldonado, 2014).

### 3.2 Data Collection and Methods

The research study was conducted in communities along coastal area vastly surrounded with bodies of water, specifically, large rivers and seas that results to extremely low temperature weather condition. The researchers selected adult clients diagnosed with CAD through purposive sampling to ensure the quality and breadth of the information needed (Ingham‐Broomfield, 2015; Sampa, 2012). The latter authors assert that exploration of a concept or phenomenon is conducted through interviews and observations with individuals who are experiencing the phenomenon. Through such, cocreation of the richness of data is achieved. Informants were chosen according to the following inclusion criteria:

- Adult clients diagnosed with CAD whose age ranges from 40 to 60 years old, both male and female.
- Adult clients diagnosed with CAD with or without medication maintenance.
- Adult clients diagnosed with CAD with or without regular medical check‐up.
- Adult clients diagnosed with CAD who have had previous cardiovascular attack like stroke with or without present paralysis and adults without CAD attack at all.
- Adult clients diagnosed with CAD who manifest interest in participating the study.

Saturation of data was achieved in 15 repeated responses from eligible informants; yet, the researchers opted to continue the interview process to 30 informants for further richness and fullness of desired quality information, as shown in Table 1.

**Table 1:** Summary of informants’ profile

Criteria	Frequency	Percentage
Age		
58–64	5	17%
49–57	15	50%
41–48	10	33%
Total	30	100%
Gender		
Male	12	40%
Female	18	60%
Total	30	100%
Maintenance intake		
With medication	17	57%
Without medication	13	43%
Total	30	100%

Standard research ethics policies and protocols were precisely observed in the entire research period. Permission letters and consent forms were submitted for approval to the office of the University of Saint Louis Graduate School, respective barangay captains and health centres in‐ charge.

The researchers utilized individual face‐to‐face interviews and observations with 30 informants (see Table 1) in investigating, exploring and gathering data on the lived experiences of the CAD‐diagnosed clients (Ingham‐Broomfield, 2015; Sampa, 2012). This serves as a research behavioural tool to encourage the people to express own feelings and individual perceptions (Malagon‐Maldonado, 2014).

Informants were individually traced with the help of barangay health worker. Structured interview was performed where responses and relative insights of the informants were jotted down; likewise, it was recorded with permission to ensure data accuracy (Malagon‐Maldonado, 2014).

### 3.3 Analysis

In this research study, Colaizzi's method (as cited in Malagon‐Maldonado, 2014; Polit & Beck, 2012) of data analysis was used. In analyzing the interview transcript reflecting the lived experiences of the informants, the researchers adhered to Colaizzi's method.

#### 3.3.1 Transcription and sorting of field notes

This initial step was completed by the researchers by repeatedly listening to the individual audio‐recorded interview session while comparing each with the field notes. This step ensured accuracy and completeness of the written verbatim transcript which facilitated succeeding steps.

#### 3.3.2 Extraction of significant statements

After transcribing all recorded interview sessions and repeatedly reading and rereading the transcription, the researchers extracted significant statements and phrases relevant to the phenomenon under the study. This step was facilitated by highlighting and underlining vital cues.

#### 3.3.3 Formulation of meanings

This step prescribed the formulation of both explicit and implicit meanings for each significant statement and cue. This involved reflection of the researchers without bias that leads to structural description of how the informants experienced the phenomenon. The researchers looked backward and forward, in parts and in whole, of the transcript to objectively immerse themselves with the lived experiences of the informants.

#### 3.3.4 Clustering

This step required the researchers to group significant statements and cues that represent the same meaning and theme. This was aided by constant rereading all cues and meanings. This substantially helped the researchers identify themes for the results presentation.

#### 3.3.5 Exhaustive description and identification

These steps in Colaizzi's method of data analysis were satisfied through reanalyzing information on the whole transcript to verify that the clustering and statements indeed incorporated the meaning of the lived experiences on CAD among the informants. These steps further involved searching for and identifying related and applicable literature which aid in the elaboration of the results of the analysis.

#### 3.3.6 Informant verification

To ensure validity, accuracy and credibility of the results of analysis, this final step required the researchers to establish rigour. This was fulfilled by presenting the results of analysis to each informant, referred to as member checking. This step significantly explicates that the analysis process done by the researchers was free from any bias or subjectivity on their part. Each informant was further asked if there is any vital information that has been omitted or that he/she wanted to substantiate. Upon satisfaction on the part of all informants, the researchers continued to write on the succeeding parts till the completion of this research study.

### 3.4 Rigour in qualitative researchers

The researchers built a trusting relationship, constant communication and member checking through the process of the study specifically in data interpretation and analysis. These were to achieve consistency, truthfulness and accuracy of the information (Malagon‐Maldonado, 2014).

### 3.5 Ethics

Prior to the actual on‐field data gathering, approved permission and secured informed consent were at hand for legality and ethicality of the study.

Informed consents were signed by the informants after discussing the research process, instructions and components of the interview, likewise, emphasizing the audio recording while the latter is ongoing. The researchers stressed out that respondents have the freedom to refuse on answering questions deemed uncomfortable or reschedule the time and place of interview at their convenience.

Informants’ privacy and information confidentiality were ensured and maintained by the researchers by the aid of alphanumeric codes. Provided personal information was kept at home of the corresponding author and was only used in study purposes and member checking or information confirmation. The document containing the list of informants and all interview transcripts and audio records in the field were destroyed at the conclusion and completion of this research study.

## 4 RESULTS

On thorough analyses of the lived experiences of adults diagnosed with CAD when intensely exposed to prolonged cold, vital information from informants was recorded (Ingham‐Broomfield, 2015; Sampa, 2012).

Using Colaizzi's method (Polit & Beck, 2012) of data analysis, three themes were identified and organized emphasizing the meanings of the collected data, namely: (i) elucidating cold exposure; (ii) challenges of cold exposure; and (iii) translating adverse exposure to self‐management.

### 4.1 Theme 1: Elucidating cold exposure

When adults diagnosed with CAD were asked about their perceptions regarding the relationship of prolonged cold exposure to their illnesses, they described them through expressing their insights to its possible effects; likewise, the causes why they are exposed to such situation. Of 30 informants, 29 shared common thoughts on the direct relationship of cold weather condition to their illness.

I04: [There might be an effect of extreme cold exposure because you get lazy to exercise, always eating hot and fatty foods and I have arthritis also, and I feel joint pains. The possible effects of extreme cold to people's health are the higher risk to hypertension due to eating and less exercise because people prefer to lie in bed. People are exposed to extreme cold due to climate change, not good shelter and not enough money to buy thick clothes as cold barrier just like I observed to our neighbour with poorly built houses.]

I10: [Yes, there is a significant relationship of cold to my illness because when the temperature is too low; my body strives very hard to cope up from the coldness. Severe coldness affects cholesterol level and fats because people prefer to sleep or not moving which results to poor circulation of the blood in the body leading to blockage in the arteries. This will cause chest pain and worst, people might suffer from heart attack when this plaques formed from fats blocked the oxygenated blood entering in and out from the heart. Eventually, intense low temperature is due to atmospheric condition or cold foods and beverages.]

I17: [There is effect of cold to me ma'am because I'm old already, of course I can feel the effect of cold: arthritis for example. When hot I can feel the effects of hypertension, but if it is cold, it does not affect a lot. I am exposed to extreme cold through fishing since it is my work to support my family]

The informants elucidated that prolonged exposure to extreme cold environmental condition, climate change, demographic location, vulnerability due to old age and poverty are associated with the development and progression of CAD. Furthermore, they commonly shared that they suffer from hypertension, joint pain, headache, cough, colds, numbness of hands and chills.

### 4.2 Theme 2: Challenges of cold exposure

Adults diagnosed with CAD narrated in full detail their personal situations, discomforts and the effects of prolonged extreme cold exposure which precisely describes individual adversities accordingly:

I03: [My body gets week when I go outside the house during cold weather]

I04: [I am exposed to cold when I get water from the bow hole far from our house. My hands get numb and paining, I am sneezing and have chest pain and it seems that there is something narrowing in my heart]

I06: [I am exposed to cold because I am taking care pigs. I have to go outside and when I take a bath, I shiver… my joints and muscles are paining because the cold permeates inside it, my nerves are constricted. When I sleep, and I wake up, my hands are numb and my muscles are cramping, now that's it is very cold, it is happening very often.]

I20: [My knees are paining, my joints, my flank, my chest palpitates strongly, I get nervous, difficulties of my disease occurs, my feet gets very cold, too]

It transpired that the informants’ prolonged exposure to cold environmental temperature is due to the nature of their work or occupation, and daily living activities even just staying at home. Complaints resulting from cold exposure included chest pain, numbness of extremities, chills, blurred vision, difficulty and shortness of breathing, palpitations, muscle stiffness/pain, dizziness, headache, cough usually with colds and joint pains.

### 4.3 Theme 3: Translating adverse exposure to self‐management

Informants consequently stated their practices and safety measures to protect themselves as their coping and adaptive methods to intense cold weather to stabilize their normal core temperature. Moreover, they cordially and confidently flaunt their pieces of advice to other people with the same health status:

I01: [In case I experience any difficulty due to cold weather which I can't tolerate, I prefer to visit the barangay health centre for medical help. It's very difficult to overcome some challenges in managing unpredictable adversities especially when no one will take care of you like me… I'm living with my son… which I am hesitant to ask assistance, for example, if I need to void. It's different to be with the care of a daughter or with a girl compared with a man]

I06: [Work so that you'll get sweat. But I get tired so I just inhale deeply. Hence, I ask help from my mother‐in‐law and sister‐in‐law. Therefore, for the people who have the same health problems, just work but take a rest if you get tired and abstain from vices and follow the doctor's advice.]

I07: [I manage it myself but sometimes I ask help from neighbourhood… It's important not to stay in cold temperature, only in places that is tolerable by the body's cold tolerance]

I19: [Avoid drinking liquor, because that is one cause of increased blood pressure, I don't have problem now my blood circulation is already okay.]

124: [I am used to self‐medication, take Aspirin that my doctor has given me and go to the nearest RHU ma'am. I'm just doing enough work Ma'am like sweeping around the house. Hence, I don't experience any difficulty because I have the full support of my family]

Respondents used warm blankets, drink warm water, eating hot or freshly cooked foods, engage in exercises and other mobility activities, drinking liquor and even smoking. For some who could not tolerate, they resort to guidance from others, such as: (i) seeking for relevant bits of advice from family and neighbours; (ii) consultation at the local health unit in their community; and (iii) consultation or confinement in the hospital. Unfortunately for handful informants, they are challenged with coping due to lack of finances for consultation, medication and/or confinement.

## 5 DISCUSSION

The study conducted through interview with 30 adults diagnosed with CAD shows that they experience adversities when they are exposed to extremely low environmental temperature for a prolonged period of time.

It was discussed by the informants that exposure to cold weather is unavoidable since they are living near the sea or river and that makes the weather very cold (Byravan & Rajan, 2015; Hamilton et al., 2015). As expected, staying outside the house and performing usual activities expose people to low temperature specifically work like fishing, farming, as barangay official and doing household chores. In addition, poor shelter and insufficient money to buy thick clothing due to poverty were emphasized; likewise, old age (Davídkovová et al., 2014; Xie et al., 2013) due to lower immune system or presence of degenerative diseases like arthritis was exemplified. The informants also knew that climate change contributes to the atmospheric condition resulting in health problems in the community (Lineman et al., 2015; Schuldt & Roh, 2014; Singh & Purohit, 2014; Zheng et al., 2012), in the case of CAD.

Consequently, people who are exposed to extreme cold of long duration encounter various adversities during prolonged extreme cold exposure that commonly associated to CAD such as musculoskeletal and respiratory manifestations (Davídkovová et al., 2014; Heaven, 2013; Lavigne et al., 2014; Schuldt & Roh, 2014; Singh & Purohit, 2014; Vosselman, Vijgen, Kingma, Brans, & MarkenLichtenbelt, 2014; Wergen et al., 2014; Zeka et al., 2014; Zhaoxing et al., 2012; Zheng et al., 2012). These commonly experienced difficulties were described by the informants as chest pain, feeling of heaviness or stabbing pain in the chest, difficulty and shortness of breath, palpitations or thready pulse, nervousness, headache, numbness of arms or hands and feet, dizziness and blurred vision which are predominant signs and symptoms of CAD. Other effects mentioned by the informants were muscle stiffness and weakness, joint pains, body pain, severe chills/shivering and cough and colds.

The effect of extreme cold exposure is unpredictable, and it varies among individuals (Asgar Pour & Yavuz, 2014; Hamilton et al., 2015; Tansey & Johnson, 2015; Xie et al., 2013; Zhaoxing et al., 2012). In this study, the highest number of cold difficulties was among female informants and older people whose age ranged from 40 to 60. In general, all the informants, both male and female, claim that they experience discomforts during intense low temperature that interfere with their health status.

Heaven (2013) and Vosselman et al. (2014) claim that when the body is exposed to extreme cold, it converts the white fats or body proteins to brown adipose tissues to produce essential extra heat to warm the body resulting in vast production of fatty acids and glycerol which may stimulate the rapid accumulation of LDL or bad cholesterol in the blood stream that promotes artery clogging leading to blockage. In addition, as the body tries to stabilize the normal core body temperature, shivering and vasoconstriction normally occur. Yet, changes to blood pressure happen affecting the arterial blood pressure such as mean blood pressure, diastolic blood pressure or fractional diastolic pressure and systolic blood pressure or fractional systolic pressure which are very important predictors of CAD (Choate, Denton, Evans, & Hodgson, 2014; Tansey & Johnson, 2015; Vosselman et al., 2014; Yu & Lumbers, 2000).

Cold stress stimulates the peripheral nervous system to activate the sympathetic response and synthesize the release of epinephrine and norepinephrine which influences cardiovascular coordination characterized by arteriolar constriction, heart rate hike and increased cardiac contractility resulting in high blood pressure that compromises CAD patients (Choate et al., 2014; Grewal, Sekhon T., Walia, & Gambhir, 2015; Silverthorn & Michael, 2013; Ze‐ Yan, et al., 2010). Davídkovová et al. (2014) and Zhaoxing et al. (2012) posit that CAD occurs when blood pressure increases through vasoconstriction and increased deposition of fats in blood vessels. These factors visibly trigger CAD that is usually manifested through direct symptoms of chest pain, palpitations, hypertension, dyspnoea and tachypnoea. The latter literature supports other manifestations experienced by the informants, such as nervousness, ease of fatigability, numbness of hands or feet, dizziness, headache and blurred vision.

Common safety measures practiced by the informants were physical protection through clothing (Tourula, Isola, Hassi, Bloigu, & Rintamäki, 2010), such as: (i) wearing a jacket, socks and multiple blankets against severe cold; (ii) drinking hot beverages like coffee and lukewarm water; and (iii) eating hot food and soup. Exercises and mobility to cope by producing body heat (Amundsen, Rognmo, Hatlen‐Rebhan, & Slordahl, 2008; Keramidas, Kölegård, Eiken, & Mekjavic, 2014) were also emphasized through body movements like dancing and vigorous working by cutting wood, sweeping the yard, cleaning the house and continuous paddling when in boat while fishing. Refraining from being exposed to cold surfaces, substances and places was also stressed by the informants. Instead, staying at home and closing windows, warming oneself near wooden fires, soaking hands and feet with warm water with salt or vinegar. Moreover, rest and medication to ease discomforts or avoid further complications were used. However, two of the informants openly shared that smoking and drinking liquor are their effective methods to combat and overcome extreme cold despite the side effect they may cause. Keramidas et al. (2014) assert that smoking and alcohol consumption are high‐risk factors that trigger dynamic cold injuries that diminish peripheral vasomotor function and may cause permanent disabilities. Cold temperature contributes to the concentration of the chemical substances of the cigarette and liquor; therefore, the effect causes further vasoconstriction blood vessels resulting to enormous damages.

Eventually, most of the informants said that they had no problems in managing cold stress because they could still manage themselves and they had a full support from their family. Nevertheless, some informants admitted struggling because they were alone, with no one to care for them or there was no female at home to assist them. A common issue had insufficient money to spend in medical consultation and to buy prescribed medicines. One informant suffered from abdominal pain while comforting herself from cold stress.

In the interview, the researchers noted that the most selected health‐care facility is the nearest local community health unit followed by the hospital if the discomforts are severe and unbearable. Some of the informants also ask assistance from neighbours or just rely on their own families. People should avoid working beyond their cold tolerance capacity and remain staying in the house or in warm areas in peak of cold season (Zhaoxing et al., 2012). It was suggested to wear jacket or thick clothing for body protection and use multiple blankets, drink hot beverages like coffee or lukewarm water, soak hands and feet to warm water and move or work to get warm. It was also important to avoid or at least reduce drinking liquor but should also focus on diet modification (Bowlin et al., 2014). Foremost, it is better to consult a health‐care practitioner for precise treatment and management (Jeffrey et al., 2015). In addition, people should be conscious of any discomforts being experienced during extreme cold which might be a sign of impending problems. Immediate medical consultation is crucial to avoid further complications, injuries and sudden death.

### 5.1 Limitation

This research study focused only on analyzing the lived experiences of adult clients diagnosed with CAD on their prolonged exposure to extreme cold. Specifically, this is limited on their perception of extreme cold and its effect to them and their self‐management activities.

## 6 CONCLUSION AND RECOMMENDATIONS

Extreme cold is a threat in CAD contributing to its development and exacerbation. This is characterized by the occurrence of the principal signs and symptoms triggered when prolonged exposed to intense low temperature. The adverse effect of this phenomenon is mostly observed and experienced in the coastal areas specifically in low‐structured places surrounded with bodies of water. Consequently, community health nursing needs to be emphasized to coastal areas to help and guide the people on the most accurate and appropriate adaptive methods regarding the phenomenon explored. It is also important to educate students about climate change and related diseases.

## CONFLICT OF INTEREST

No conflict of interest has been declared by the authors.
